# Mesenchymal stem cells inhibit lipopolysaccharide-induced inflammatory responses of BV2 microglial cells through TSG-6

**DOI:** 10.1186/1742-2094-11-135

**Published:** 2014-08-04

**Authors:** Yi Liu, Run Zhang, Ke Yan, Fanfan Chen, Weiyi Huang, Bingke Lv, Chengmei Sun, Limin Xu, Feng Li, Xiaodan Jiang

**Affiliations:** 1 The National Key Clinic Specialty, the Neurosurgery Institute of Guangdong Province, Guangdong Provincial Key Laboratory on Brain Function Repair and Regeneration, Department of Neurosurgery, Zhujiang Hospital, Southern Medical University, 253# Gongye Road, Guangzhou 510282, China

## Abstract

Microglia are the primary immunocompetent cells in brain tissue and microglia-mediated inflammation is associated with the pathogenesis of various neuronal disorders. Recently, many studies have shown that mesenchymal stem cells (MSCs) display a remarkable ability to modulate inflammatory and immune responses through the release of a variety of bioactive molecules, thereby protecting the central nervous system. Previously, we reported that MSCs have the ability to modulate inflammatory responses in a traumatic brain injury model and that the potential mechanisms may be partially attributed to upregulated TNF-α stimulated gene/protein 6 (TSG-6) expression. However, whether TSG-6 exerts an anti-inflammatory effect by affecting microglia is not fully understood. In this study, we investigated the anti-inflammatory effects of MSCs and TSG-6 in an *in vitro* lipopolysaccharide (LPS)-induced BV2 microglial activation model. We found that MSCs and TSG-6 significantly inhibited the expression of pro-inflammatory mediators in activated microglia. However, MSC effects on microglia were attenuated when TSG-6 expression was silenced. In addition, we found that the activation of nuclear factor (NF)-κB and mitogen-activated protein kinase (MAPK) pathways in LPS-stimulated BV2 microglial cells was significantly inhibited by TSG-6. Furthermore, we found that the presence of CD44 in BV2 microglial cells was essential for MSC- and TSG-6-mediated inhibition of pro-inflammatory gene expression and of NF-κB and MAPK activation in BV2 microglial cells. The results of this study suggest that MSCs can modulate microglia activation through TSG-6 and that TSG-6 attenuates the inflammatory cascade in activated microglia. Our study indicates that novel mechanisms are responsible for the immunomodulatory effect of MSCs on microglia and that MSCs, as well as TSG-6, might be promising therapeutic agents for the treatment of neurotraumatic injuries or neuroinflammatory diseases associated with microglial activation.

## Introduction

Microglia, which are derived from primitive myeloid progenitor cells, are the resident immune cells of the central nervous system (CNS) [1]. As CNS-specific macrophages, they play a critical role in immune surveillance and homeostatic maintenance of the CNS [2]. They are highly responsive to stress and injury and become immediately and focally activated in response to brain injuries, systemic infections, and chronic diseases [3,4]. Furthermore, microglia are involved in initiating inflammatory responses in the brain through secreting a variety of inflammatory mediators, including tumor necrosis factor (TNF)-α, interleukin (IL)-1β, IL-6, and nitric oxide (NO) [5,6]. Many recent studies have attributed neuronal damage to the inflammatory responses of the microglia rather than to a direct neurotoxic insult [7,8]. Reactive microglia are a common feature of numerous types of brain pathology [9]. Therefore, modulating microglia function and activity appears to be an attractive approach to treating CNS injuries.

Mesenchymal stem cells (MSCs) are a population of heterogeneous multipotent cells that reside primarily in bone marrow but can be found in various postnatal organs and tissues, such as adipose tissue [10], umbilical cord blood [11], and amniotic fluid [12]. These cells are relatively easy to isolate, expand rapidly in culture, and differentiate into several cellular phenotypes *in vitro* and *in vivo*. Due to their self-renewal and potential multilineage properties, MSCs appear to be an ideal cellular source for the repair of CNS injuries. Previous studies have tested the therapeutic potential of MSCs in treating CNS injuries, including traumatic brain injury (TBI), stroke, and spinal cord injury in animal models. The results of these studies suggested that transplanted MSCs have beneficial effects on CNS injuries. For instance, MSCs may promote functional neurological recovery, decrease apoptosis levels, foster endogenous neurogenesis, improve angiogenesis, and reduce lesion size [13].

The initial assumption in exploring the mechanisms by which MSCs ameliorate CNS injury was that they migrated to the injured tissues and transdifferentiated to replace damaged neural cells [13,14]. However, recent studies showed that transplanted MSCs exert their therapeutic effect without evidence of engraftment [15,16], which indicated that their regenerative and differentiating abilities may not play a role in enhancing tissue repair or limiting tissue destruction. Instead, MSCs that are stimulated via an inflammatory signal secrete a variety of bioactive molecules, such as trophic factors and anti-inflammatory molecules, to modulate the host microenvironment [17], which may be the main mechanism responsible for their therapeutic effects [18].

We recently showed that MSCs can modulate inflammation-associated cytokine release and immune cell activation during TBI-induced cerebral inflammatory responses. We also showed that the beneficial effects of MSCs may be partially explained by the effect of TSG-6 on the nuclear factor (NF)-κB pathway [19]. However, whether TSG-6 exerts an anti-inflammatory effect by directly affecting the resident inflammatory cells of the CNS (mainly microglial cells) remains unclear.

To understand the mechanism of the effects of MSCs on neuroinflammation induced by microglia, we used the *in vitro* lipopolysaccharide (LPS)-induced BV2 microglial activation model. Changes in the expression of pro-inflammatory mediators and in members of intracellular signaling pathways by microglia in response to the LPS stimulus were evaluated. The results indicated that TSG-6 expression by MSCs plays a vital role in suppressing LPS-induced overactivation of BV2-microglial cells. Moreover, CD44 expression by microglial cells is essential for the TSG-6-induced downregulation of LPS and TLR4-mediated signal transduction.

## Materials and methods

### Ethics statement

Six-to-eight-week-old female BALB/c mice were purchased from the Laboratory Animal Center of the Southern Medical University (Guangzhou, China). All animal experiments were conducted in accordance with the National Institutes of Health Guide for the Care and Use of Laboratory Animals (National Institutes of Health publication number 80-23, 1996 revision). All experimental procedures were approved by the Southern Medical University Ethics Committee.

### Mesenchymal stem cells isolation and culture

MSCs were prepared from mouse bone marrow (BM) cells, as previously described [20], with minor modifications. MSCs were isolated from the BM of the tibias and femurs of male BALB/c mice. The cells were plated in 25-cm^2^ culture flasks containing Dulbecco’s modified Eagle medium (DMEM; Gibco, Grand Island, New York, United States) supplemented with 10% heat-inactivated fetal bovine serum (FBS; Gibco), 10 nM GlutaMAX-I supplement (Invitrogen, Raritan, New Jersey, United States), 100 U/ml penicillin, 100 μg/ml streptomycin (Hyclone, Logan, Utah, United States), and 10 μg/ml gentamycin (Hyclone) and were grown at 37°C in a humidified 5% CO2 atmosphere. After a 48-hour incubation, the non-adherent cells were removed and fresh culture medium was added to the flasks. One-half of the medium was replaced twice a week. When the cells reached 80 to 90% confluence, adherent cells were trypsinized, harvested, and expanded. MSC were assessed using flow cytometry to detect cells that expressed the typical markers CD106, CD44, and Sca-1, and that were negative for CD11b, CD45, and MHC-II (Additional file 1: Figure S1). The MSCs used in the experiments were from passages 6 to 8.

### Activation of mesenchymal stem cells using TNF-α

Before being utilized in co-cultures, MSCs were activated using TNF-α, as described previously [21]. In brief, MSCs were plated at 2 × 10^5^ cells per well in 6-well plates containing 2 mL of DMEM with 10% heat-inactivated FBS and were incubated for 1 day. The medium was then changed to DMEM containing 2% heat-inactivated FBS and 10 ng/mL TNF-α (R&D Systems, Minneapolis, Minnesota, United States). After incubation for 18 hours, the cells were trypsinized using 0.25% trypsin (Gibco) for 3 minutes at 37°C. To confirm the increased expression of TSG-6, RNA was extracted from cell aliquots (TRIzol™, Invitrogen) and assayed for TSG-6 expression using real-time RT-PCR (Additional file 2: Figure S2).

### Transfection of mesenchymal stem cells with TSG-6 siRNA

A total of 2 × 10^5^ MSCs were plated in 6-well dishes and cultured for 24 hours. The cells were then transfected with TSG-6 siRNA or control siRNA (sc-39820, sc-37007; Santa Cruz Biotechnology Inc, Paso Robles, California, United States) using Lipofectamine™ 2000 according to the manufacturer’s instructions (Invitrogen). To confirm the silencing effect of the TSG-6 siRNA, RNA was extracted from aliquots of the cells after 48 hours and analyzed for TSG-6 expression using real-time RT-PCR (Additional file 3: Figure S3).

### BV2 cell culture

The BV2 murine microglial cell line was obtained from Xiehe Medical University (Beijing, China). The cells were cultured in a humidified incubator at 37°C with 5% CO2 in DMEM supplemented with 10% heat-inactivated FBS, 100 μg/ml streptomycin, 100 U/ml penicillin (Hyclone), and 2 mmol/l glutamine (Invitrogen) at 37°C in a humidified atmosphere containing 5% CO2. A 6-well transwell system (0.3-mm pore size membrane; Corning, Cambridge, Massachusetts, United States) was used to assess the effect of MSCs on BV2 cells that were stimulated using LPS. A total of 5 × 10^5^ BV2 cells were placed in the lower chamber and stimulated with or without 100 ng/mL of LPS in the presence of one of the following treatments in the upper chamber (recombinant mouse TSG-6 protein(rmTSG-6) was added directly to the microglial cultures during the LPS treatment): 1) control medium, 2) 1.0 × 10^5^ TNF-α-activated MSCs, 3) 1.0 × 10^5^ activated MSCs transfected with TSG-6 siRNA, 4) 1.0 × 10^5^ activated MSCs transfected with control siRNA, 5) rmTSG-6 at 1 ng/ml, 6) rmTSG-6 at 10 ng/ml, or 7) rmTSG-6 at 100 ng/ml.

### RNA isolation and quantification real-time polymerase chain reaction (qRT-PCR)

After 6 hours of treatment, the expression levels of TNF-α, IL-1β, IL-6, and induced nitric oxide synthase (iNOS) mRNA in BV2 cells were evaluated using real-time PCR. In brief, total RNA was isolated from BV2 cells using TRIzol™ reagent (Invitrogen) according to the manufacturers’ instructions. Total RNA from each sample was reverse transcribed using oligo dT and SuperScript III RT (Invitrogen). qRT-PCR was conducted using an ABI 7500HT rapid real-time PCR system (Applied Biosystems, Foster City, California, United States). Glyceraldehyde-3-phosphate dehydrogenase (GAPDH) was employed as the endogenous control. The sequence-specific primers used for the above genes are listed in Additional file 4: Table S1. The 2^-ΔΔCt^ method was employed to calculate relative expression levels.

### Western blotting analysis

Western blotting was performed to assess whether TSG-6 affected the downstream TLR4 signaling pathways of the BV2 cells. LPS-activated BV2 cells were cultured in the presence of 10 ng/ml rmTSG-6 (R&D Systems) for 15, 30, or 60 minutes, and total proteins were extracted using RIPA lysis buffer (sc-24948; Santa Cruz Biotechnology, California, United States). Protein concentrations were estimated using a bicinchoninic acid (BCA) protein assay kit (Thermo Scientific, Pierce, Rockford, Illinois, United States). The proteins were separated by sodium dodecyl sulfate-polyacrylamide gel electrophoresis and transferred to polyvinylidene difluoride membranes (Millipore, Billerica, Massachusetts, United States). The membranes were blocked using 5% non-fat dry milk in Tris-Buffered Saline and Tween 20 (TBST) and then incubated with primary antibodies directed against JNK, p38, Erk, phospho-JNK, phospho-p38 or phospho-Erk (Cell Signaling Technology, Danvers, Massachusetts, United States) at a 1:1000 dilution at 4°C overnight. The secondary antibodies were applied for 1 hour at room temperature. The immunoblots were visualized using enhanced chemiluminescence (ECL; Thermo Scientific). The cellular expression levels were normalized to GAPDH (Cell Signaling Technology).

### Immunofluorescence and laser-scanning confocal microscopy

Immunofluorescence staining and laser-scanning confocal microscopy were performed as previously described. BV2 cells were grown on poly-L-lysine- (Sigma, St Louis, MO, USA) coated glass slides for 12 hours. BV2 cells were stimulated using LPS in the presence of 10 ng/ml rmTSG-6 for 1 hour. After treatment, the cells were washed using PBS and fixed using 4% paraformaldehyde at room temperature. The cells were exposed to a primary antibody directed against NF-κB p65 (rabbit monoclonal NF-κB p65 antibody, 1:200) at 4°C overnight. Following primary antibody incubation, the slides were incubated with the secondary antibody (Alexa Fluor 594 goat anti-rabbit IgG (1:200)) for 1 hour at room temperature. After three washes using PBS, the slides were incubated with 5 μg/ml FITC-Phalloidin (Sigma) for 20 minutes at room temperature. The slides were mounted using ProLong® Gold Antifade Reagent containing 4', 6-diamidino-2-phenylindole (DAPI, Invitrogen). Images were obtained using an Olympus FV1000 confocal microscope (Olympus, Tokyo, Japan) and analyzed using FV10-ASW 3.0 Viewer software (Olympus). The percentage of cells with NF-κB p65 staining localized to the nucleus was determined by examining at least 100 cells per slide.

### Electrophoretic mobility shift assay (EMSA)

To examine the binding of NF-κB to DNA, nuclear extracts were prepared using NE-PER Nuclear and Cytoplasmic Extraction Reagents (Thermo Scientific). Synthetic complementary NF-κB-binding oligonucleotides (5′-AGT TGAGGG GAC TTT CCC AGG C-3′) (Santa Cruz Biotechnology) were biotinylated using a biotin 3′-DNA labeling kit (Thermo Scientific) according to the manufacturer’s instructions. The binding reactions were conducted for 20 minutes at room temperature in the presence of 50 ng/ml poly(dI-dC), 0.05% Nonidet P-40, 5 mM MgCl2, 10 mM ethylene diamine tetraacetic acid (EDTA), and 2.5% glycerol in 1 × binding buffer (LightShift chemiluminescent EMSA kit, Thermo Scientific) with 20 fmol biotin-end-labeled target DNA and 10 μg of nuclear extract. The samples were loaded onto 6.5% non-denaturing polyacrylamide gels pre-electrophoresed in 0.5 × Tris/Boric Acid/EDTA (TBE) buffer at 100 V for 60 minutes and were electrophoresed in 0.5 × TBE buffer at 100 V for 45 minutes. Then, the samples were transferred to a positively charged nylon membrane (Millipore) in 0.5 × TBE on ice at 390 mA for 30 minutes. The transferred DNAs were cross-linked to the membrane at 120 mJ/cm^2^ and detected using horseradish peroxidase-conjugated streptavidin (Thermo Scientific) according to the manufacturer’s instructions.

### Transient transfection of NF-κB reporter construct and luciferase assay

The level of transcriptional activity of the NF-κB gene was measured using an NF-κB luciferase reporter gene assay (Promega, Madison, Wisconsin, United States). Briefly, BV2 cells were seeded at a density of 1 × 10^5^ in 24-well plates and co-transfected with 0.2 μg of the pNF-κB-luciferase reporter plasmid (Bayotime, Shanghai, China) and 0.02 μg of the pRL-TK control plasmid (Promega) using Lipofectamine™ LTX (Invitrogen) according to the manufacturer’s protocol. After 24 hours, the culture medium was replaced with DMEM, and LPS stimulation was conducted in the presence of MSCs or TSG-6. Luciferase activity levels were determined using the Dual-Luciferase reporter Assay System (Promega) and normalized to that of *Renilla reniformis*. The altered luciferase activity was described as the relative ratio to the control activity.

### Transfection of BV2 cells with CD44 siRNA

To knockdown CD44 expression, BV2 cells were transfected with CD44 siRNA or control siRNA (sc-35534, sc-37007; Santa Cruz Biotechnology) using Lipofectamine™ 2000 (Invitrogen) according to the manufacturer’s instructions. After 48 hours, CD44 knockdown was assessed using western blotting and immunofluorescence assays.

### Statistical analyses

All experiments were performed at least three times, and the data are expressed as the mean value ± SD. Statistical analyses were performed using SPSS 13.0 software (SPSS, Chicago, Illinois, United States). A one-way analysis of variance (ANOVA) was used to determine the significance of differences between the groups. Significance was set at a level of *P* <0.05.

## Results

### Mesenchymal stem cells and TSG-6 suppressed the lipopolysaccharide-induced pro-inflammatory gene expression of microglia

**Figure 1:**
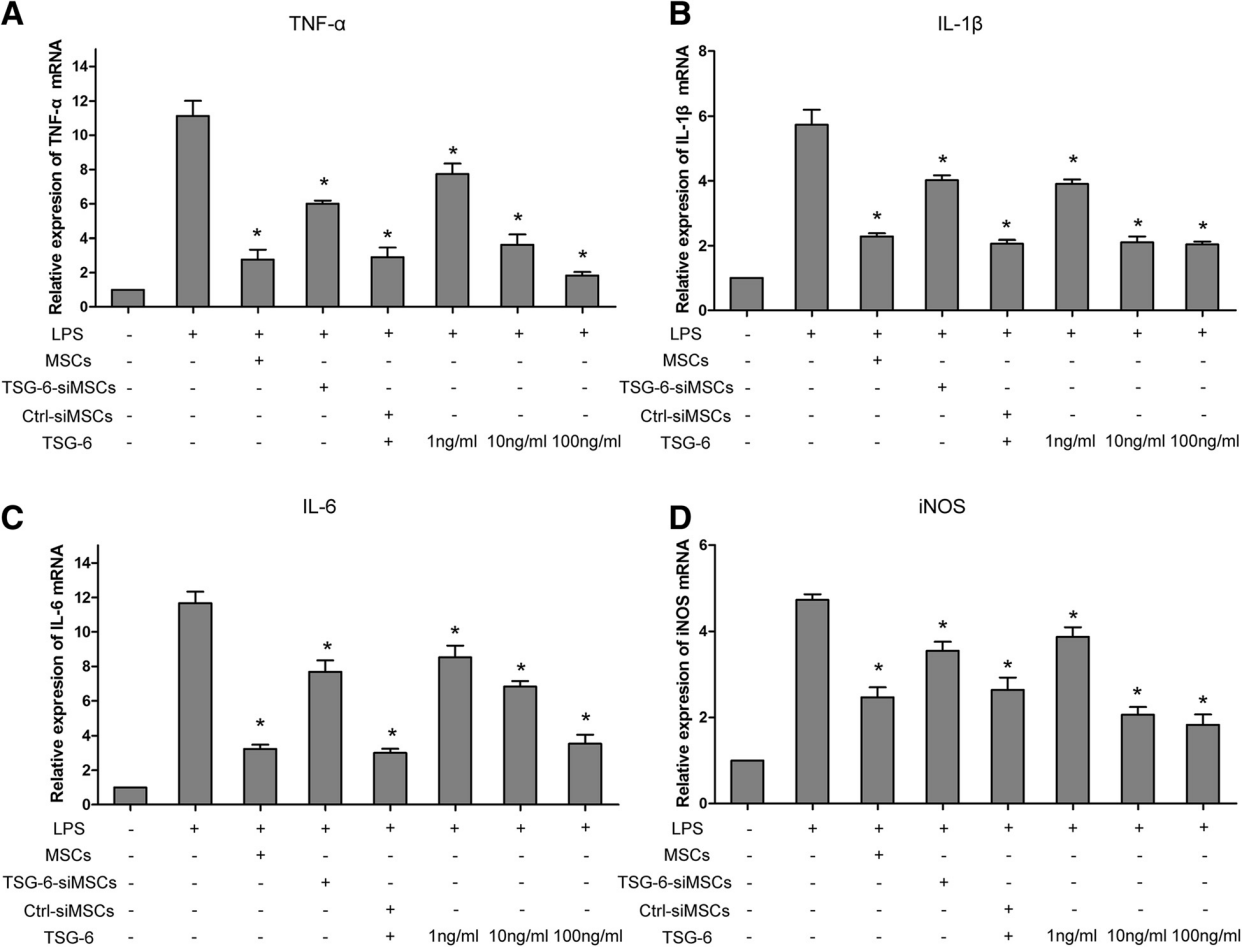
**MSCs and TSG-6 suppress LPS-induced pro-inflammation gene expression of microglia.** BV2 cells were co-cultured with MSCs, TSG-6-siMSCs, or control-siMSCs activated by TNF-α and different concentration of rmTSG-6 (1 ng/ml, 10 ng/ml, 100 ng/ml) and treated with or without LPS (100 ng/ml) for 6 hours. **(A-D)**, mRNA levels of TNF-α, IL-1β, IL-6, and iNOS were measured by qRT-PCR analyses and quantified as fold induction over the levels in unstimulated control cells. All transcriptional levels were normalized to GAPDH mRNA levels and determined by the 2^-ΔΔCt^ method. Values in the bar graphs are presented as mean ± SD. n = 3; *P <0.05; significantly different from LPS-treated cells. Abbreviations: TSG-6-siMSCs, MSCs transfected with TSG-6 siRNA; Ctrl(control)-siMSCs, MSCs transfected with control siRNA; LPS, lipopolysaccharide; iNOS, induced nitric oxide synthase.

We first investigated whether the mRNA expression of the anti-inflammatory gene TSG-6 was increased in MSCs that had been stimulated by the inflammatory cytokine TNF-α. Using qRT-PCR analysis, we found that in response to TNF-α stimulation, MSCs expressed TSG-6 mRNA to a level seven-fold higher than the control level (Additional file 2: Figure S2). Next, we investigated the effect of MSCs and TSG-6 on the expression of inflammatory factors by LPS-activated microglia. To this end, BV2 microglial cells were co-cultured with MSCs and rmTSG-6 at different concentrations for 6 hours. As shown in Figure 1, we observed a significant decrease in the levels of TNF-α, IL-1β, IL-6, and iNOS mRNA expression when LPS-stimulated BV2 cells were cultured in the presence of MSCs. However, the MSCs had reduced effects when the expression of the TSG-6 gene was knocked down using TSG-6 siRNA compared with non-transfected MSCs or control siRNA transfected MSCs. To confirm the key role of TSG-6 in modulating the function and activity of the LPS-activated microglia, we added rmTSG-6 directly to the microglial cultures during LPS treatment. TSG-6 supplementation largely reproduced the effects of the MSCs in a dose-dependent manner. The results shown in Figure 1 demonstrate that in the presence of 10 ng/ml and 100 ng/ml of TSG-6, the levels of TNF-α, IL-1β, IL-6, and iNOS mRNA expression by the BV2 microglia were significantly reduced, similar to those produced by activated MSCs.

### TSG-6 interfered with the lipopolysaccharide-induced activation of NF-κB signaling

**Figure 2:**
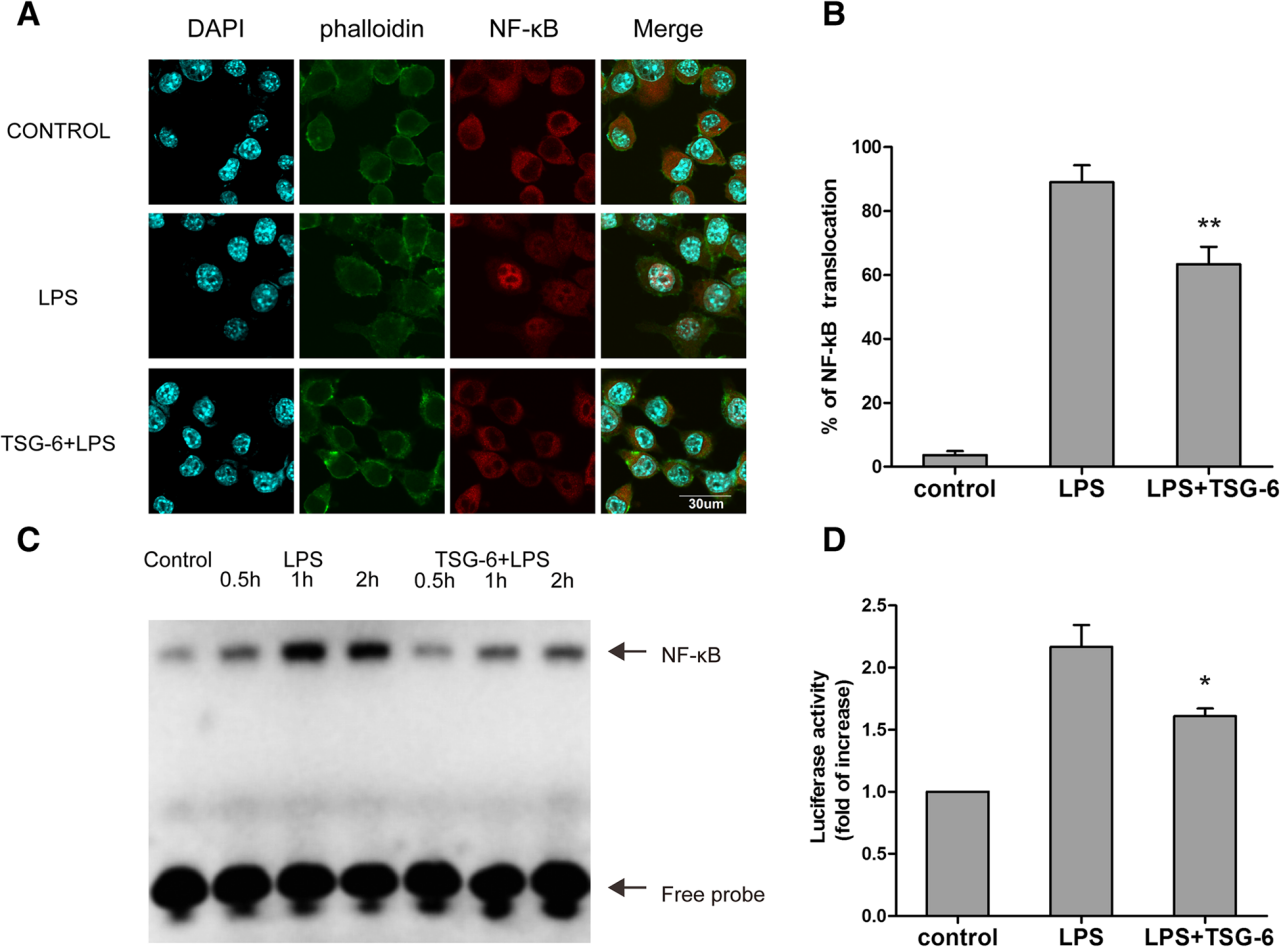
**TSG-6 interferes with LPS-induced activation of NF-κB signaling.** BV2 cells were stimulated with LPS in the presence and absence of TSG-6 (10 ng/ml). Cells were immunostained with a primary antibody against NF-κB p65, followed by an Alexa Fluor 594-conjugated secondary antibody. Actin filaments (green) and cell nuclei (blue) were visualized with FITC-labeled phalloidin and DAPI separately. Cell images were obtained using a confocal microscopy. **(A)** Typical micrographs of immunocytochemistry are shown for cytoplasmic and nuclear distribution on NF-κB p65. Scale bars, 30 μm. **(B)** The percentages of cells with NF-κB p65 localized to the nucleus were determined by analysis of at least 100 cells per slide. **(C)** Nuclear extracts were prepared and processed for chemiluminescence-based NF-κB EMSA experiments. Cells were stimulated with 100 ng/ml LPS with or without TSG-6(10 ng/ml) for the indicated periods. Nuclear extracts were incubated with a biotin-labeled NF-κB-specific oligonucleotide and further probed with streptavidin-HRP. The arrow indicates shifted DNA probe for NF-κB and free probe respectively. **(D)** BV2 cells were co-transfected with pNF-κB-luciferase reporter plasmid and pRL-TK control plasmid and then treated with or without LPS (100 ng/ml) in appearance or absence of rmTSG-6 (10 ng/ml) for 6 hours. NF-κB activities were measured by luciferase assay, normalized to luciferase activities of pRL-TK, and quantified as fold changes over the control (unstimulated BV2 cells). Values are mean ± SD. n = 3; **P <0.01; *P <0.05; significantly different from LPS-treated cells. Abbreviations: LPS, lipopolysaccharide; NF-κB, nuclear factor (NF)-κB.

The above results indicate that TSG-6 was involved in reducing the level of LPS-mediated expression of inflammatory mediators in BV2 cells. Thus, we investigated whether TSG-6 played a role in the inflammatory signaling events triggered by LPS stimulation. Because NF-κB is a major transcription factor mediating pro-inflammatory gene expression in the LPS-TLR4 signaling pathway [22], we examined possible alterations in NF-κB signaling induced by TSG-6 in BV2 microglia. First, we analyzed NF-κB p65 nuclear translocation in BV2 cells using immunofluorescence staining and confocal laser-scanning microscopy. After a 1-hour treatment, the cells were fixed for immunostaining. NF-κB p65 largely remained in the cytoplasm of the control group under basal (unstimulated) conditions. Upon LPS stimulation, nuclear translocation of NF-κB p65 occurred in BV2 cells, but to a lesser extent in the TSG-6-treated group (Figure 2A, B).We next evaluated the DNA-binding activity of NF-κB using a chemiluminescence-based EMSA assay. Nuclear extracts were prepared 0.5, 1, and 2 hours after LPS stimulation of control and TSG-6-treated cells, and were used to compare the binding of a biotin-labeled NF-κB-specific DNA probe . The increase in DNA-protein complex formation was less prominent as early as 0.5 hours after LPS stimulation in the TSG-6-treated cells than in the LPS-treated cells (Figure 2C).Finally, we evaluated NF-κB transcriptional activity using luciferase reporter assays. pNF-κB-Luc containing five tandem repeats of an NF-κB consensus binding site or pRL-TK as the normalization control were transiently expressed for 24 hours before LPS stimulation. Measurements of the luminescence intensity showed that TSG-6 inhibited LPS-induced NF-κB activation (Figure 2D). Taken together, the results suggested that TSG-6 was actively involved in the regulation of TLR4-mediated NF-κB signaling.

### TSG-6 attenuated the lipopolysaccharide-mediated phosphorylation of p38, JNK and Erk mitogen-activated protein kinases

**Figure 3:**
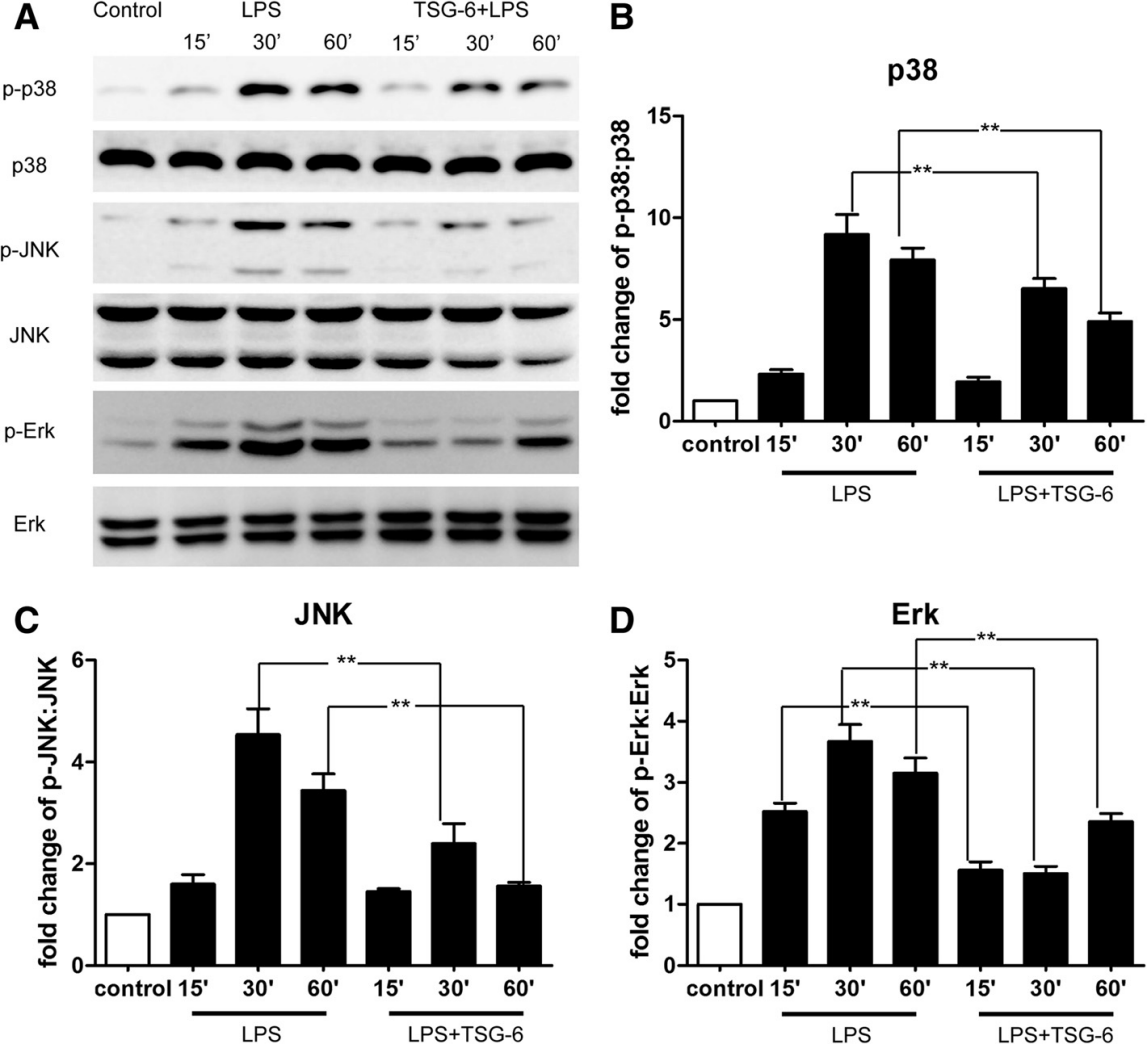
**TSG-6 suppresses LPS-mediated phosphorylation of p38, JNK, and Erk.** BV2 cells were stimulated with LPS in the presence and absence of rmTSG-6 (10 ng/ml) for the indicated times. Cell lysates were analyzed for the phosphorylated levels of p38, JNK, Erk, and their total levels by western blotting with the respective specific antibodies. **(A)** Representative image of protein levels of P38, JNK, and Erk. **(B-D)** The phospho:total ratios of p38 **(B)**, JNK **(C)**, and Erk **(D)** were determined by measurements of band intensities of each protein kinase and quantified as fold changes over the control (unstimulated BV2 cells). X axis are shown in time slots of 15, 30, and 60 minutes. Values are mean ± SD. n = 3; **P <0.01; significantly different from LPS-treated cells. Abbreviations: LPS, lipopolysaccharide.

Members of the mitogen-activated protein kinase (MAPK) family, such as p38, JNK, and Erk, are important downstream effector molecules that participate in TLR4 signaling, which can mediate pro-inflammatory gene expression [23]. Therefore, we examined whether TSG-6 might also disrupt MAPK signaling. BV2 cells were stimulated with LPS in the presence of 10 ng/ml rmTSG-6 for 15, 30, or 60 minutes. The level of activation of the MAPKs was then assessed by measuring the levels of phosphorylated p38, JNK, and Erk using western blotting. As shown in Figure 3A-D, The levels of phosphorylated p38 and JNK were slightly increased after 15 minutes of treatment in the LPS and LPS + TSG-6 groups, whereas the differences between the levels of LPS-induced phosphorylation of p38 and JNK in the two groups were less remarkable. The levels of phosphorylated p38 MAPK and JNK were significantly increased after 30 to 60 minutes of LPS stimulation compared to those of the control cells, whereas the LPS-induced phosphorylation of both kinases was relatively attenuated in TSG-6-treated BV2 cells at 30 and 60 minutes post-stimulation. Phosphorylated Erk levels increased significantly as early as 15 minutes after LPS stimulation compared with that of the control cells. However, the LPS-induced phosphorylation of Erk was significantly reduced in the presence of TSG-6 at 15, 30, and 60 minutes post-stimulation. Taken together, these results suggest that TSG-6 treatment of microglia downregulated LPS/TLR4-mediated MAPK activation.

### The inhibitory effects of TSG-6 were dependent on CD44

**Figure 4:**
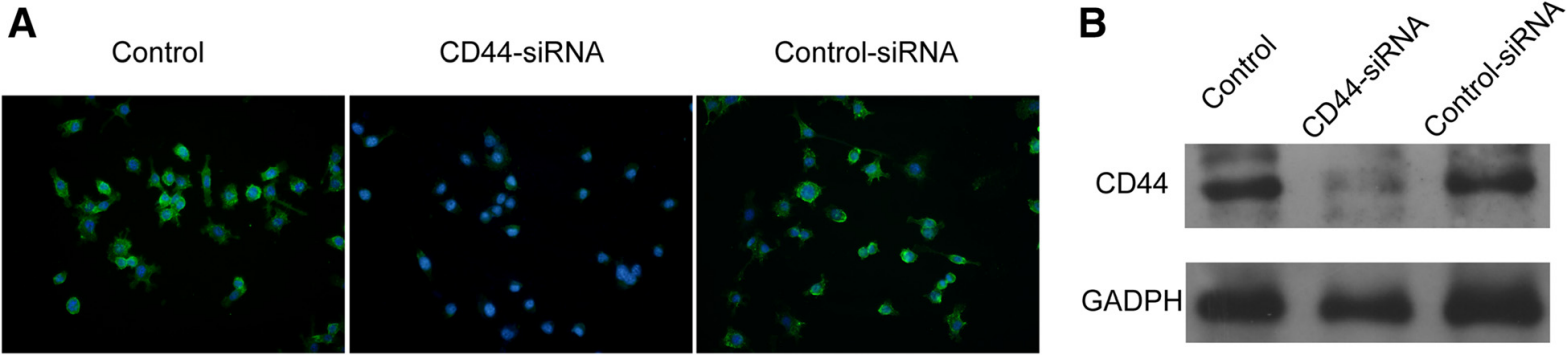
**siRNA-mediated downregulation of CD44 expression in BV2 cells. (A)** Representative immunofluorescence images of CD44 (green) in control BV2 cells, CD44-siRNA-BV2 cells, and control-siRNA-BV2 cells. Nuclei are stained with DAPI (blue). Magnification × 400. **(B)** Western blot assay was performed to determine CD44 protein expression in extracts of control BV2 cells, CD44-siRNA-BV2 cells, and control-siRNA-BV2 cells. GAPDH was used as a loading control. Abbreviations: CD44-siRNA-BV2, BV2 cells transfected with CD44 siRNA; control-siRNA-BV2, BV2 cells transfected with control siRNA, DAPI, 4', 6-diamidino-2-phenylindole.

**Figure 5:**
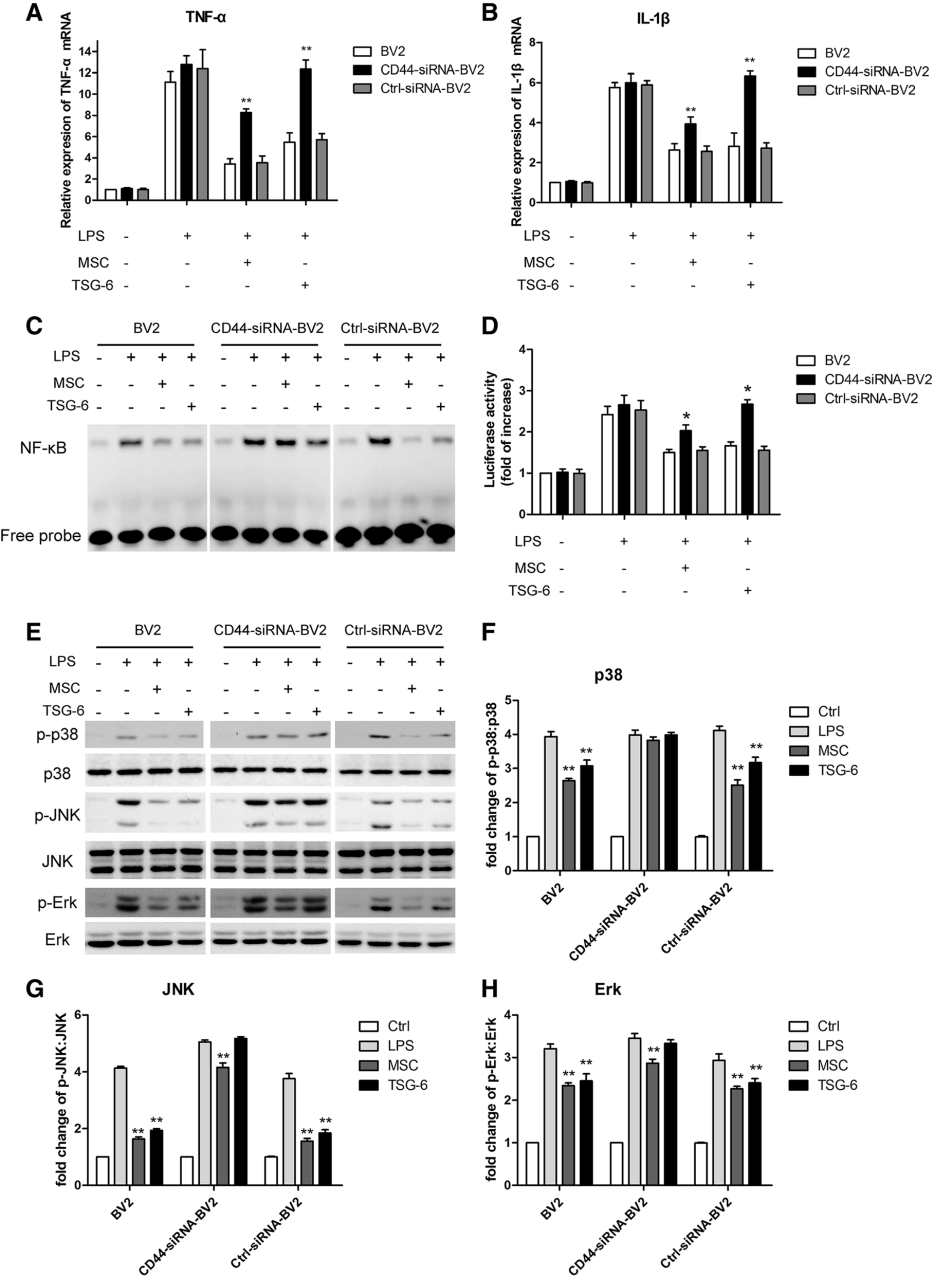
**CD44 was essential for the MSC- and TSG-6-mediated anti-inflammatory responses. (A, B)** BV2 cells, CD44-siRNA-BV2 cells, and control-siRNA-BV2 cells were incubated for 6 hours with LPS and with or without activated MSCs or TSG-6. The mRNA levels of TNF-α and IL-1β were measured by qRT-PCR. All transcriptional levels were normalized to GAPDH mRNA levels and determined by the 2^-ΔΔCt^ method. Values are mean ± SD. n = 3; **P <0.01; significantly different from control BV2 cells or control-siRNA-BV2 cells. **(C)** NF-κB DNA-binding activities were measured by EMSA assay. BV2 cells, CD44-siRNA-BV2 cells, and control-siRNA-BV2 cells were incubated for 1 hour with LPS and with or without activated MSCs or TSG-6. The data are representative of at least three independent experiments. **(D)** Luciferase reporter assays for NF-κB transcriptional activity in BV2 cells, CD44-siRNA-BV2 cells, and control-siRNA-BV2 cells (conditions as in Figure 2D) Values are mean ± SD. n = 3; *P <0.05; significantly different from control BV2 cells or control-siRNA-BV2 cells. **(E)** Cell extracts were prepared from BV2 cells, CD44-siRNA-BV2 cells, and control-siRNA-BV2 cells incubated for 30 minutes with LPS and with or without activated MSCs or TSG-6, then subjected to immunoblot analysis using antibodies against the phospho- or total-forms of the three MAPKs. **(F-H)** Quantification of western blot data. The phospho:total ratios of p38 **(F)**, JNK **(G)**, and Erk **(H)** were determined by measurements of band intensities of each protein kinase and quantified as fold changes over the control (unstimulated BV2 cells). Values are mean ± SD. n = 3; **P <0.01; significantly different from LPS-treated cells. Abbreviations: CD44-siRNA-BV2, BV2 cells transfected with CD44 siRNA; control-siRNA-BV2, BV2 cells transfected with control siRNA; LPS, lipopolysaccharide; NF-κB, nuclear factor (NF)-Κb; EMSA, electrophoretic mobility shift assay.

A previous study demonstrated that the inhibitory effects of TSG-6 on zymosan-induced TLR2/NF-kB signaling activation were dependent on the expression of CD44 on macrophages. Furthermore, CD44 suppressed NF-κB activation after LPS stimulation [21]. TSG-6 also can inhibit the activation of both T-cells and antigen-presenting cells (APCs) in a CD44-dependent manner [24]. To obtain insights into the role of CD44 and the relationship between CD44 and TSG-6 in modulating the activities of LPS-activated microglia, we repeated some of the above-reported experiments using BV2 cells in which CD44 expression was knocked down using CD44 siRNA. The efficiency of the CD44 knockdown was confirmed using western blotting and immunofluorescence. The expression of CD44 was significantly reduced (Figure 4A, B). As shown in Figure 5A and B, the presence of MSCs or rmTSG-6 had less effect on reducing the expression of the pro-inflammatory cytokines TNF-α and IL-1β in CD44-knockdown BV2 cells compared with non-transfected control cells or control-siRNA-transfected cells. The magnitude of the LPS-induced increased DNA-binding activity of NF-κB, as well as the degree of NF-κB transcriptional activity, was significantly higher in CD44-siRNA-transfected BV2 cells than in non-transfected control cells or control-siRNA-transfected cells in the presence of MSCs or rmTSG-6. (Figure 5C, D) Consistent with these observations, the western blotting results showed that MSCs or rmTSG-6 had substantially less impact on reducing the phosphorylation levels of p38 (Figure 5E, F), JNK (Figure 5E, G) and Erk (Figure 5E, H) MAPKs upon LPS stimulation in CD44-knockdown BV2 cells compared with wild-type non-transfected control cells or control-siRNA-transfected cells. (Figure 5C, D) These results suggest that CD44 is required for the MSC- and TSG-6-mediated anti-inflammatory responses.

## Discussion

The aims of this study were to determine whether TSG-6 exerted an anti-inflammatory effect by modulating microglia function and to examine the possible underlying mechanisms. Therefore, we used an *in vitro* LPS-stimulated BV2 microglial model to investigate changes that occurred in pro-inflammatory mediator expression and in members of inflammatory signaling pathways in the presence of MSCs or TSG-6. In this study, we found that MSCs and TSG-6 mediated the LPS-induced inflammatory responses of microglia. Our results clearly showed that MSCs and TSG-6 reduced the LPS-induced expression of TNF-α, IL-1β, IL-6, and iNOS. In experiments designed to elucidate the molecular mechanism underlying the anti-inflammatory function of TSG-6 in microglia, we found that TSG-6 attenuated the LPS-induced activation of NF-κB signaling and the phosphorylation of p38, JNK, and Erk Moreover, the anti-inflammatory effect of TSG-6 depended on CD44 expression on BV2 microglial cells.

Microglia are activated by various stresses and by brain injury in order to provide supportive signals that lead to neuronal recovery. However, excessive activation of microglia, characterized by robust production and secretion of a variety of pro-inflammatory mediators, results in neuronal injury which is associated with the pathogenesis of a number of neurological disorders such as multiple sclerosis, trauma, Parkinson’s disease, and Alzheimer’s disease [25,26]. Thus, a therapeutic approach targeting activated microglia may be a promising treatment for these conditions.

Recently, many studies have demonstrated that MSCs possess immunomodulatory properties. MSCs can directly inhibit the proliferation of T and B lymphocytes [27,28], inhibit dendritic cell maturation and function [29], and modulate the cytokine-secretion profile of monocytes and macrophages [30]. MSCs are also known to inhibit the basal and formyl-methionyl-leucyl-phenylalanine-stimulated production of reactive oxygen species by neutrophils [31]. Due to these immunosuppressive properties, MSC-based therapy has been successfully applied to various immune-related diseases, such as graft-versus-host disease (GvHD) [32], systemic lupus erythematosus (SLE) [33], experimental autoimmune encephalomyelitis [34], and multiple sclerosis (MS) [35].

Previously, we reported that MSCs have the ability to modulate inflammatory responses in a TBI model and that the potential mechanisms responsible for the anti-inflammatory effects of MSCs may be partially explained by upregulated TSG-6 expression. TSG-6, also known as TNAIP6, is a 30 kD glycoprotein that is expressed at substantially higher levels by many cell types (including MSCs) in response to stimulation by several pro-inflammatory mediators compared to normal physiological conditions [36]. Several studies have shown that TSG-6 produces anti-inflammatory effects both *in vivo* and *in vitro *[15,16,37]. In the present study, we found that TSG-6 was expressed at a substantially higher level in MSCs stimulated by TNF-α. The TNF-α-activated MSCs were co-cultured with BV2 cells followed by LPS stimulation. MSCs significantly reduced the level of pro-inflammatory gene expression in BV2 cells. However, when we utilized MSCs that were transfected with siRNA directed against TSG-6 in the co-cultures with activated microglia, we detected a partial restoration of the expression level of those genes compared with that of LPS-stimulated BV2 cells in the presence of non-transfected MSCs. To identify the functional roles of TSG-6 in microglial inflammatory responses induced by LPS, we added recombinant TSG-6 protein directly to microglial cultures during LPS treatment. The recombinant TSG-6 protein also reduced the expression of the pro-inflammatory genes in a dose-dependent manner. These data demonstrated that TSG-6 has a key role in MSC-mediated modulation of LPS-activated microglia.

LPS induces microglial activation through the expression of TLR4, which triggers the association of myeloid differentiation factor 88 (MyD88)-independent and -dependent pathways. More importantly, the MyD88-dependent pathway sequentially activates a cascade of enzymes and transcription factors, including NF-κB and MAPKs, such as p38, JNK, and Erk [23].

NF-κB is a critical transcription factor that regulates the expression of various pro-inflammatory genes. NF-κB normally exists in the cytosol under unstimulated conditions, bound to the inhibitory IκB protein. LPS stimulation leads to an increase in the nuclear translocation of NF-κB and in its DNA-binding activity at specific promoter regions of a variety of pro-inflammatory genes such as iNOS, TNF-α, IL-1β, and IL-6, which modulates their expression [22]. To elucidate the molecular mechanism underlying the anti-inflammatory function of TSG-6 in microglia, we first evaluated its effect on NF-κB activity. The results demonstrated that LPS-induced nuclear translocation, DNA-binding activity, and transcriptional activity of NF-κB in BV2 cells were substantially suppressed by TSG-6. In addition to the NF-κB pathway, the MAPK pathway was found to be associated with the LPS-induced activation of BV2 microglial cells [38,39]. Some studies have suggested that MAPKs are crucial for the LPS-stimulated expression of iNOS, COX-2, and TNF-α [40]. Our study showed that LPS increased the activation of p38, JNK, and Erk in BV2 cells. However, pretreatment with TSG-6 significantly decreased the level of activation of these kinases in LPS-stimulated microglia. More importantly, we found that the inhibitory effects of TSG-6 were dependent on CD44 expression on microglial cells. Because MSCs or TSG-6 inhibited pro-inflammatory gene expression, NF-κB and MAPK activation was significantly restored in CD44-knockdown BV2 cells after LPS stimulation. These results are consistent with previous observations that the inhibitory effects of TSG-6 on NF-кB signaling were dependent on CD44 expression on macrophages and that TSG-6 might regulate the activity of TLR pathways upstream of MyD88 [21]. TSG-6 is capable of binding to hyaluronan (HA), moreover, TSG-6 and HA have been reported to form stable complexes that modulate the interaction of HA with the cell surface receptor CD44 [41], which is involved in TLR4 signaling through the formation of a TLR4-CD44 complex. One study has shown that the engagement of CD44 by HA suppressed TLR4 signaling [42]. In addition, there is an increasing body of evidence showing that CD44 is a negative regulator of TLR2- and TLR4-mediated inflammation [43]. Because crosslinking HA with TSG-6 can significantly alter the affinity of CD44 for HA, TSG-6 may stabilize the binding of CD44 to HA [44], which are in close proximity to TLR4, thus hindering the binding of TLR4 agonists and thereby blocking TLR4 signaling. However, our study does not definitively support such a mechanism, and thus this hypothesis is only speculative. Further research must be performed to determine the specific mechanisms of action. Another limitation of our article is the lack of demonstration of the findings in primary microglia, although BV2 microglial cells are commonly used as an alternative to primary microglia to study various microglial responses and interactions [20,45,46]. To fully understand the anti-inflammatory and immunomodulatory properties of TSG-6, further experiments should focus on the role of TSG-6 on primary microglia.

In summary, we have demonstrated that following stimulation with TNF-α, MSCs modulate microglia activity through TSG-6. TSG-6 may interact with CD44 on microglial cells to inhibit LPS-induced activation of TLR4 signaling, decreasing NF-κB activity and the activation of MAPK signaling, thus diminishing the levels of expression of neuroinflammatory cytokines in BV2 microglia. These results are consistent with the recent evidence that MSCs can inhibit inflammation through diffusible molecules and that stimulation with certain inflammatory cytokines is essential for MSC-mediated immunosuppression. Several soluble factors have been reported to be involved in MSC-mediated immunoregulation, such as PGE2, IDO [47], and CX3CL1 [48], which are released by MSCs following stimulation with inflammatory factors. Therefore, in addition to TSG-6, it is apparent that MSCs can produce a variety of anti-inflammatory factors and the results presented here do not rule out the effects of other anti-inflammatory factors. However, the data obtained in experiments using TSG-6 siRNA and rmTSG-6 demonstrates that TSG-6 might play a vital role in inhibiting LPS-stimulated microglial activation.

## Conclusions

The results of this study suggest that MSCs mediate microglial activation and the production of inflammatory factors through TSG-6. Our data suggest novel mechanisms for the MSC-mediated immunosuppression of microglia. These findings can not only serve as the basis for future studies of the beneficial immunomodulatory effects of MSCs but may also promote the future clinical application of MSCs to neurotraumatic and/or neuroinflammatory disorders in which microglia may play a major pathogenic role.

## Abbreviations

MSC: Mesenchymal stem cell; TBI: Traumatic brain injury; CNS: Central nervous system; TSG-6: TNF-α stimulated gene/protein 6; NF-κB: Nuclear factor-κB; EMSA: electrophoretic mobility shift assay; LPS: lipopolysaccharide.

## Competing interests

The authors declare that they have no competing interests.

## Authors’ contributions

Conceived and designed the experiments: YL, XDJ. Performed the experiments: YL, RZ, KY, FFC, WYH, CMS, FL. Analyzed the data: LMX, BKL. Wrote the paper: YL, XDJ. Paper revision: XDJ. All authors read and approved the final manuscript.

## Supplementary Material

Additional file 1: Figure S1 Surface marker expression in MSCs. MSCs were confirmed by flow cytometry analysis after three passages as positive for CD44 (98.60%), CD106 (88.28%), and Sca-1 (98.28%), with low positivity for CD11b (0.98%), CD45 (0.64%), and MHC-II (0.55%). Click here for file

Additional file 2: Figure S2 MSCs overexpress TSG-6 mRNA in response to inflammatory cytokine TNF-α. Relative expression levels of TSG-6 mRNA was determined by qRT-PCR. **P <0.01 versus control. Click here for file

Additional file 3: Figure S3 The expression of the TSG-6 gene was knocked down with using TSG-6 siRNA. Relative expression levels of TSG-6 mRNA was determined by qRT-PCR. **P <0.01 versus control or control siRNA. Click here for file

Additional file 4: Table S1 Primers used in this study. Click here for file
