# Supplementary figures and images for: Mesenchymal stem cells inhibit lipopolysaccharide-induced inflammatory responses of BV2 microglial cells through TSG-6
Source: J Neuroinflammation. 2014 Aug 4;11:135. doi: 10.1186/1742-2094-11-135 (PMC4128538; doi:10.1186/1742-2094-11-135)

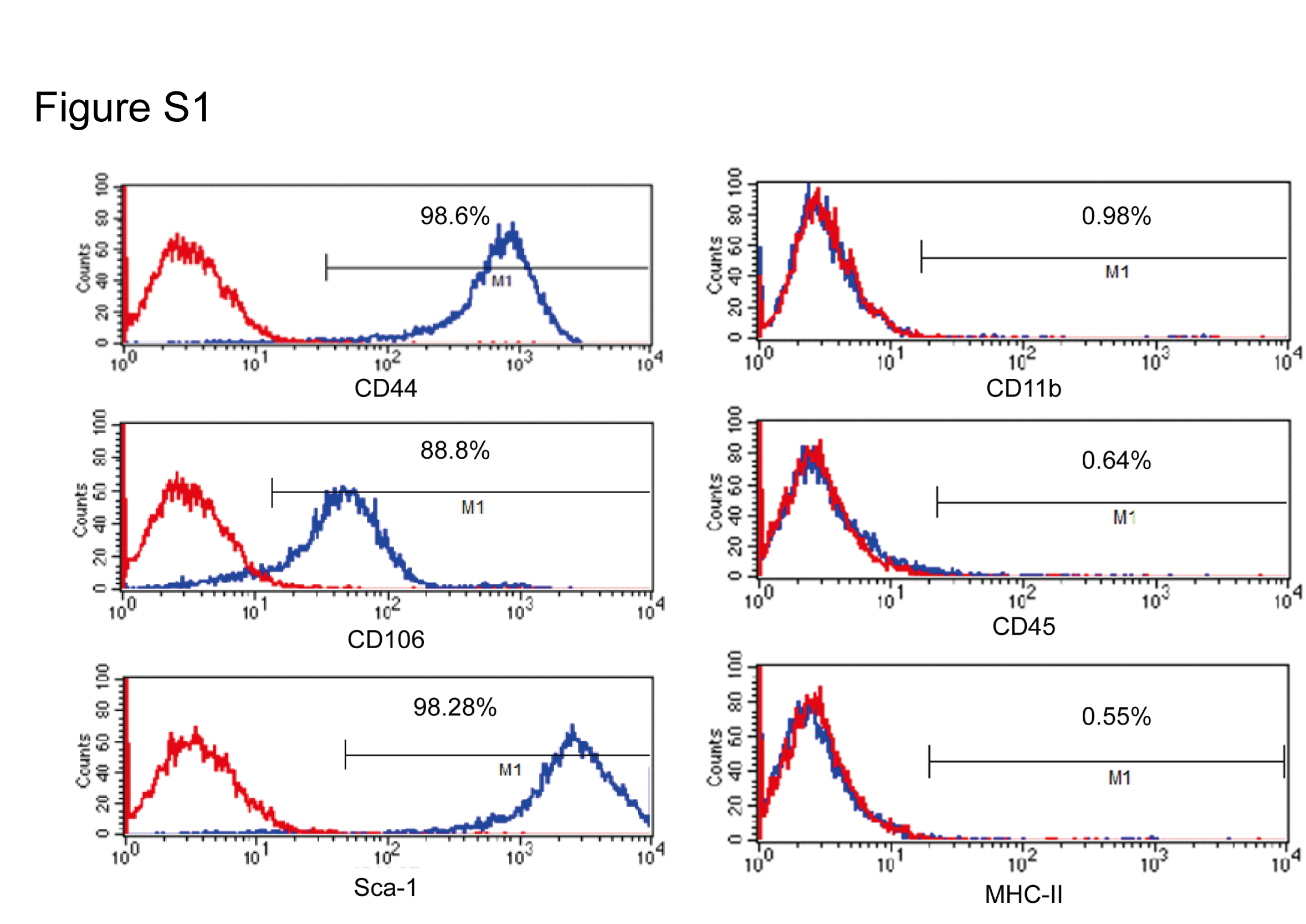

Supplement: Additional file 1: Figure S1 — Surface marker expression in MSCs. MSCs were confirmed by flow cytometry analysis after three passages as positive for CD44 (98.60%), CD106 (88.28%), and Sca-1 (98.28%), with low positivity for CD11b (0.98%), CD45 (0.64%), and MHC-II (0.55%). [file 1742-2094-11-135-S1.tiff]

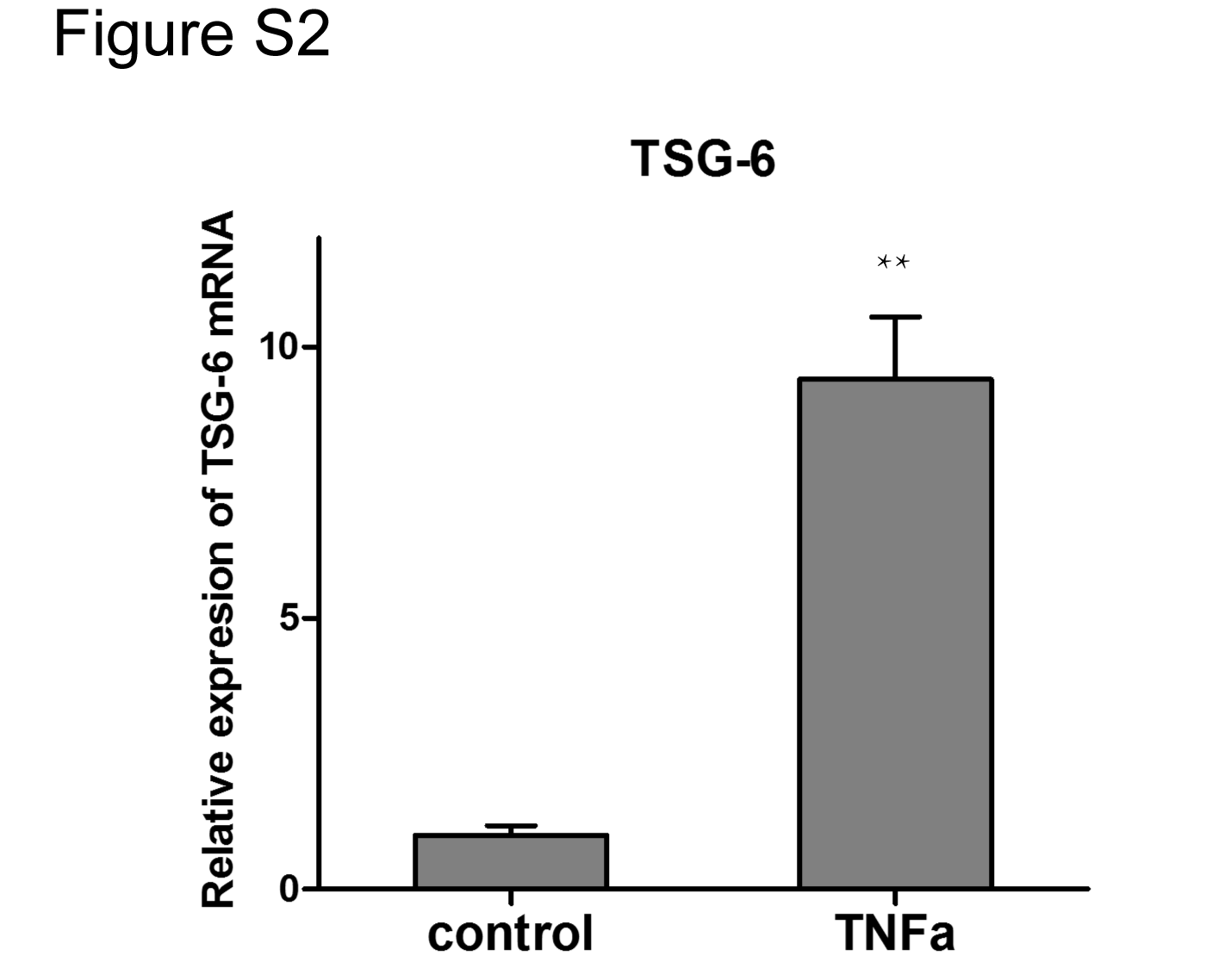

Supplement: Additional file 2: Figure S2 — MSCs overexpress TSG-6 mRNA in response to inflammatory cytokine TNF-α. Relative expression levels of TSG-6 mRNA was determined by qRT-PCR. **P <0.01 versus control. [file 1742-2094-11-135-S2.tiff]

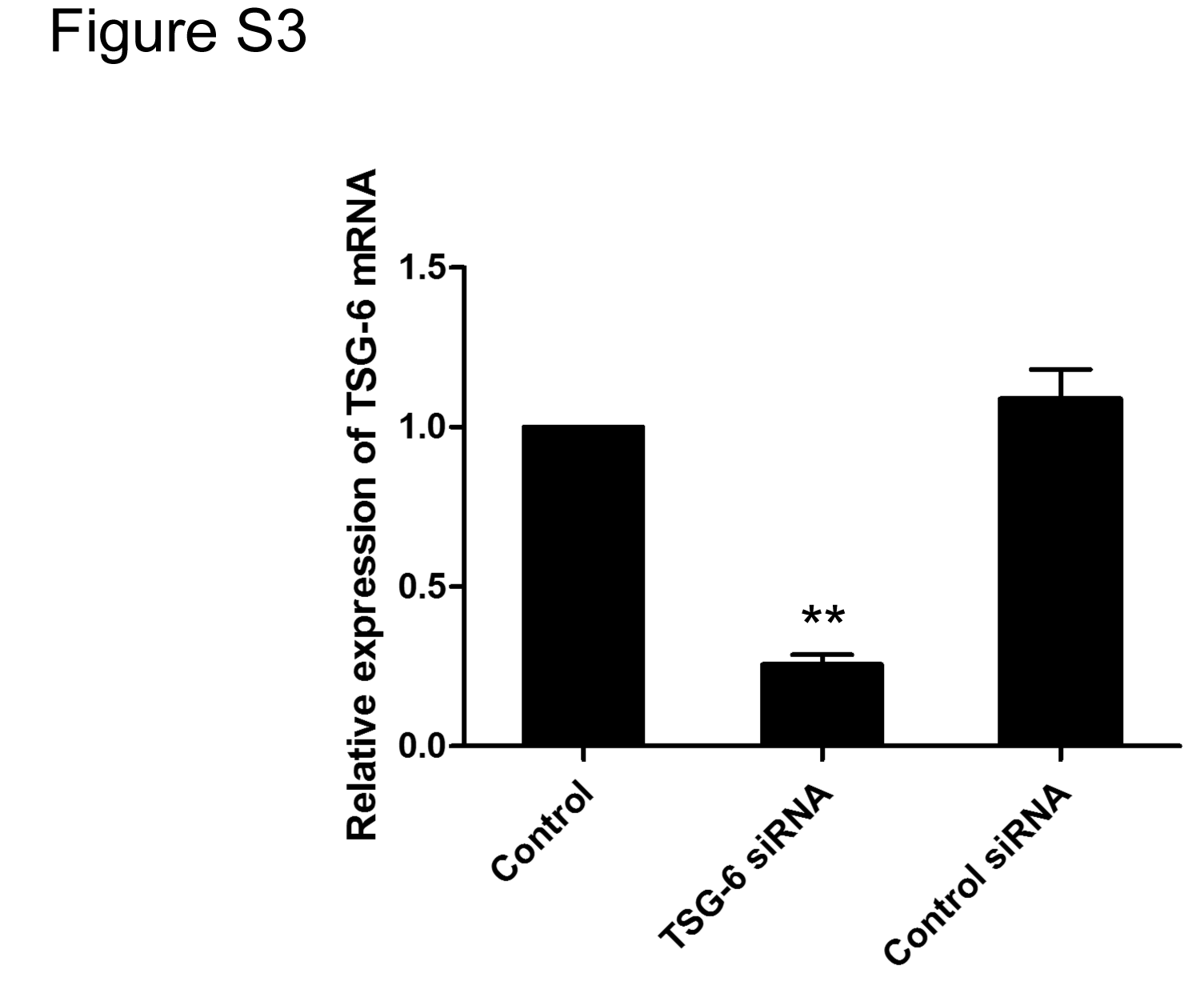

Supplement: Additional file 3: Figure S3 — The expression of the TSG-6 gene was knocked down with using TSG-6 siRNA. Relative expression levels of TSG-6 mRNA was determined by qRT-PCR. **P <0.01 versus control or control siRNA. [file 1742-2094-11-135-S3.tiff]
